# The Role of Constraint in Revision Total Knee Replacement for Instability: Full Component Revision Vs Isolated Polyethylene Exchange in Selected Patients

**DOI:** 10.1016/j.artd.2023.101134

**Published:** 2023-04-25

**Authors:** Nicolas Sapountzis, Vignesh K. Alamanda, Chisa Hidaka, Amethia Joseph, Yu-fen Chiu, Michael Cross, José A. Rodríguez

**Affiliations:** a Adult Reconstruction and Joint Replacement, Hospital for Special Surgery, New York, NY, USA; b Biostatistics Core, Hospital for Special for Surgery, New York, NY, USA

**Keywords:** Knee revision, Isolated polyethylene exchange, Constraints, Outcomes research, TKA instability

## Abstract

**Background:**

Instability is a common indication for revision after total knee arthroplasty. Replacement of multiple components is the current standard, but isolated polyethylene liner exchange (IPE) may present a less-morbid alternative. This study aims to determine (1) whether IPE results in similar rerevision frequency to component revision in select patients with symptomatic instability and (2) the effect of increasing constraint on the outcome.

**Methods:**

We retrospectively reviewed 117 patients revised for symptomatic total knee arthroplasty instability from January 2016 to December 2017. The component revision (60 patients) or IPE (57 patients) cohorts were further stratified based on whether constraint was increased or not. The primary objective was to compare rerevision rates 2 years after component revision vs IPE. The secondary objectives consisted of evaluating reasons for rerevision, preoperative and postoperative patient-reported outcome measures, and range of motion.

**Results:**

The rerevision rate was 18%, with no statistical difference between component and IPE cohorts. Cases where level of constraint increased due to revision, a significantly lower rate of rerevision was detected (9 of 77) (12%) than in cases where constraint did not increase (12 of 39) (31%) (P=0.012). This association was also noted in the component revision cohort but not in the IPE cohort (P=0.011).

**Conclusions:**

Rerevision occurred at similar frequencies 2 years after IPE or component revision for total knee arthroplasty instability. For component revision, increased constraint was associated with significantly fewer rerevisions.

## Introduction

Instability remains a common cause of early failure after total knee arthroplasty (TKA), accounting for 10%-22% of revision TKAs [[1], [2], [3], [4], [5], [6], [7]]. The gold standard for treatment of TKA instability is a one or both component revision with optimization of alignment of components and equalization of flexion and extension gaps, but this type of revision surgery is complex and recovery can be prolonged [5,7]. Previous studies have reported that isolated polyethylene exchange (IPE), an alternative with lower associated morbidity, can be as successful as component revision in select patients with rerevision rates as low as 6.3% when constraint is increased [[8], [9], [10], [11], [12]]. However, the use of IPE to treat TKA instability remains controversial with some groups reporting rerevision rates as high as 60% [[13], [14], [15]]. Regardless of the overall failure rates within a given study, some patients do well with IPE, and our goal is to identify which patients these may be. Understanding the true efficacy and ideal indications for IPE can limit inappropriate use of this technique where it is unlikely to provide benefit. Conversely, the advantages of IPE, including shorter operating times, less blood loss, preservation of bone stock, shorter and less-complicated recovery, as well as lower hospital costs [16] can only be realized when appropriately indicated.

In this study, we retrospectively reviewed consecutive cases of revision TKA performed for instability at our institution to determine the proportion of patients undergoing IPE and document their knee alignment. We then compared their outcomes to those of the patients undergoing one or both component revision. Rerevision was the primary end point. We also assessed knee range of motion (ROM) and patient-reported outcomes measures including Knee Injury and Osteoarthritis Outcome (KOOS JR), Lower Extremity Activity Scale (LEAS), and pain visual analog scale (VAS).

## Material and methods

After institutional review board approval, a retrospective review of a prospectively collected data from a total joint registry at a single major healthcare system was conducted. Consecutive patients who underwent a unilateral revision total knee arthroplasty for a diagnosis of instability (diagnosis codes: T84.022A and T84.023A) from January 2016 to December 2017 were identified. We defined instability as a state of perceived dysfunction reported by the patient and observed assessment of laxity by the surgeon. Patients who were revised for additional reasons such as wear, loosening, or periprosthetic joint infection were excluded. We also excluded patients with prior revision history, less than 2-year minimum follow-up, or unknown rerevision status. However, any rerevised patient from our initial cohort between January 2016 and December 2017 were included regardless of prior revision history, 2-year minimum follow-up, or unknown rerevision status.

Patients who underwent revision with isolated polyethylene insert exchange (IPE) were identified based on chart review. The distal femoral valgus angle, proximal tibial cut angle, lateral tibial slope, and overall limb alignment were measured on these patients’ prerevision radiographs to make certain that they did not have gross bony malalignment or compromised fixation and were thus appropriate candidates for IPE.

Outcomes of patients undergoing IPE were then compared to those of the rest of the cohort, who underwent revision of either tibial, femoral, or both components (component revision). Each group of patients (component revision and IPE) was further stratified based on whether the level of constraint was increased during revision. Constraint was categorized into 4 levels – (1) Lowest, which included cruciate retaining implants, (2) Low, which included posterior stabilized implants, (3) High, which included posterior stabilized constrained and varus valgus constrained implants, and (4) Highest, a linked rotating hinge. Any degree of change to a higher level of constraint was designated as “increased,” and outcomes were compared between patients with or without an increase in level of constraint.

Demographic information including age, body mass index, laterality, and race was collected. In addition, length of stay, and discharge disposition were recorded. The primary outcome was frequency of rerevision for any reason, and indications for rerevision were documented. To measure patient functional status, we compared preoperative and postoperative ranges of motion, which were measured during in clinic follow-up at least 1 year after revision TKA. We also compared patient-reported outcome measures at minimum 2 years. The patient-reported outcome measures included KOOS JR, LEAS, and pain VAS.

Continuous variables were reported as mean +standard deviation, and categorical variables were presented as frequencies and percentages. Chi-squared and t-tests were used, and all tests were 2-sided. Significance was defined as *P* < .05, and Excel and SAS were used to perform statistical analysis.

## Results

**Figure 1:**
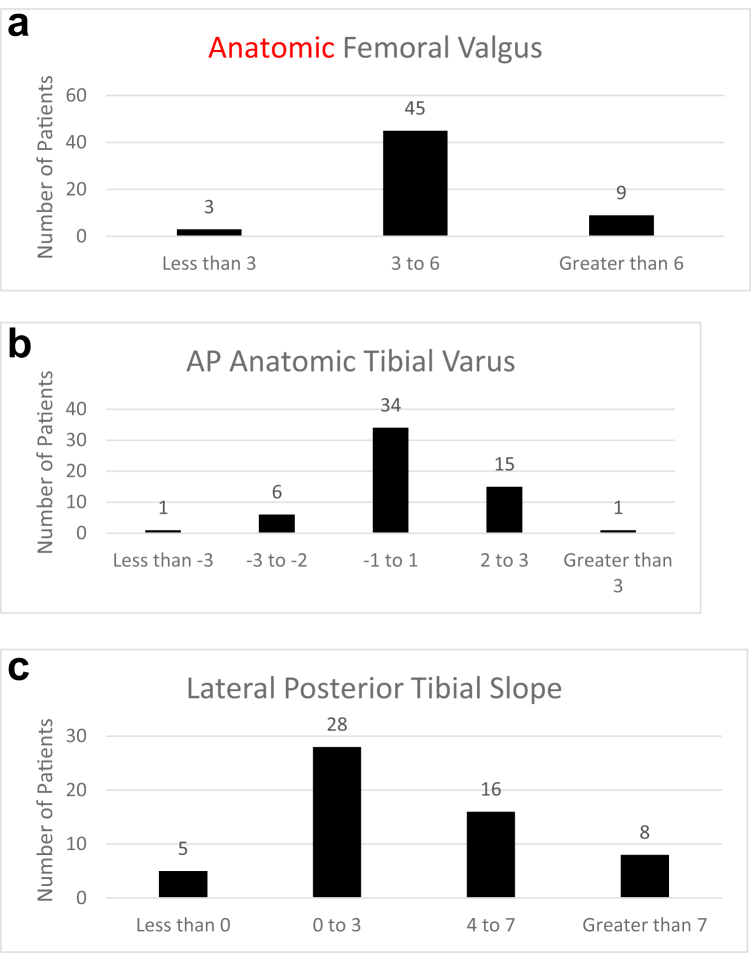
Radiographic parameters of knee alignment in patients who underwent isolated polyethylene exchange, (a) The anatomic distal femoral angle was measured using radiographic scans of an anteroposterior “AP” view of the knee. Alignments were in the normal range, between 3 and 6 degrees of valgus, in 45 (79%) of IPE patients. (b) The anatomic tibial angle was measured using radiographic scans of an anteroposterior “AP” view of the knee. Normal alignment, within 1 degree of mechanical alignment with respect to varus, was observed in all but 2 of 57 patients undergoing IPE. (c) The Posterior Tibial Slope was measured using radiographic scans of a lateral view of the knee. The slope was between 0 and 7 degrees in 44/57 (77%) of IPE patients. AP, anterior-posterior; IPE, isolated polyethylene exchange.

Of 117 patients whose TKAs were revised for instability, 57 (49%) underwent isolated IPE. We confirmed that these patients had been appropriately selected for IPE based on radiographs showing no gross bony malalignment (Fig. 1a-c). In the majority of IPE patients, anterior-posterior anatomic distal femoral angle were cut at approximately 3 to 6 degrees of valgus with respect to their anatomic axis (Fig. 1a). The anterior-posterior proximal tibia cut angle in the majority of patients was within 1 degree of the neutral mechanical axis (Fig. 1b). Most patients had 0-7 degrees of posterior slope (Fig. 1c).

Patients who underwent IPE were not significantly different from the rest of the cohort, who underwent component revision (N = 60; 51%) with regard to demographic characteristics such as age, sex, body mass index, and race (Table 1). Laterality, length of stay, discharge disposition, and mean follow-up were also similar between groups. The IPE cohort was more likely to have had their primary knee replacement performed at the author’s institution compared to the component revision cohort (*P* = .0006). The polyethylene insert thickness increased from about 11 to 15 mm in both the groups (*P* = .633).

**Table 1:** Demographic characteristics of patients undergoing component revision or isolated polyethylene insert exchange (IPE) for unstable total knee arthroplasty.

Demographic variable	Component revision (N = 60)		IPE (N = 57)		*P*-value
	Mean	Standard deviation	Mean	Standard deviation	
Age (y)	66	9.4	66	7.7	.806
BMI (kg/m^2^)^a^	31.1	6.7	29.8	7.4	.339
Length of stay (d)	3.3	1.1	2.9	2.0	.243
Time to last follow-up (y)	3	1.2	3	1.1	.452
Polyethylene insert size (mm)					
Preoperative^a^	11	2.6	11	3.0	.980
Postoperative	16	4.4	16	3.9	.549
delta^a^	4	4.7	4	3.1	.784
	N	Percent	N	Percent	
Primary surgery at HSS					.0010
unknown	2	3	0	0	
No	35	58	17	30	
Yes	23	39	40	70	
Side					.811
Right	35	58	32	56	
Sex					.895
Female	38	63	35	61	
Race					.118
Unknown	2	3	0	0	
Asian	1	2	1	2	
Black or African American	7	12	5	9	
White or Caucasian	44	73	49	86	
Other	6	10	2	4	
Discharge disposition					.133
Home	3	5	10	18	
Home w/ home health	42	70	38	67	
Rehabilitation facility/unite	2	3	2	4	
Skilled nursing facility	13	22	7	12	

a Body mass index (BMI) was available for 59 component revision and 51 IPE patients. Preoperative polyethylene insert size and delta (difference in) preoperative and postoperative insert size was available for 44 component revision and 51 IPE patients.

Rerevision frequency was similar following component revision (N = 10, 17%) and IPE (N = 11, 19%) (Table 2, *P* = .711). In both the groups, the most common reasons for rerevision were infection and instability. Instability accounted for 35% of overall rerevision cases and was more frequent in the IPE group (N = 5, 9%) than in the component revision patients (N = 2, 3%).

**Table 2:** Frequency and reasons for rerevision following component revision or isolated polyethylene insert exchange (IPE) for unstable total knee arthroplasty.

Variable	Component revision (N = 60)		IPE (N = 57)		*P*-value
	N	%	N	%	
Rerevision	10	17	11	19	.711
Reason for rerevision					
Infection	3	5	2	3	
Instability	2	3	5	9	
Loosening	4	7	2	3	
Malalignment	1	2	0	0	
Polydislocation	0	0	1	2	
Osteolysis	0	0	1	2	

To determine whether a change in the level of constraint affected revision TKA outcomes, we stratified each group into 2 categories: increased or not increased, as defined in Methods. The number and proportion of patients, receiving each type of implant, based on level of constraint are reported in Table 3. Overall, revision surgery increased the level of constraint in a greater number of patients who underwent component revision (N = 47, 78%) than IPE (N = 30, 53%) (*P* = .002, Table 4). In the component revision group, 18 patients (31%) had the lowest level of constraint (cruciate retaining), and none had the highest level (hinge knee in place) prior to their revision surgery. After revision, 50 patients (85%) in the component revision group had a high level of constraint (posterior stabilized constrained or varus valgus constrained) and 6 patients (10%) had the highest level (hinge). Increases in the level of constraint were also noted in the IPE group in which the number and proportion of patients with a low level of constraint (posterior stabilized implant) decreased from 45 patients (79%) to 15 (26%), while those with a high level of constraint (posterior stabilized constrained or varus valgus constrained) increased from 9 patients (16%) to 39 patients (68%) upon revision surgery.

**Table 3:** Implant type and constraint level before and after component revision or isolated polyethylene insert exchange (IPE)^a^.

Level of constraint	Implant type:	Component revision N = 60		IPE N = 57	
		Preoperative N (%)	Postoperative N (%)	Preoperative N (%)	Postoperative N (%)
Lowest	Cruciate retaining	18 (31%)	0 (0%)	3 (5%)	3 (5%)
Low	Posterior stabilized	23 (39%)	4 (7%)	45 (79%)	15 (26%)
High	Posterior stabilized constrained	7 (12%)	10 (17%)	4 (7%)	8 (14%)
	Varus valgus constrained	11 (19%)	40 (68%)	5 (9%)	31 (54%)
Highest	Hinge	0 (0%)	6 (10%)	0 (0%)	0 (0%)

a Constraint level was considered to have increased if the postoperative level of constraint of the patient’s knee implant was higher than the preoperative level, as described in Methods.

**Table 4:** Change in constraint after component revision or isolated polyethylene insert exchange (IPE) for unstable total knee arthroplasty.

Constraint	Component revision N = 60	IPE N = 57	*P* value
Increase in level of constraint at revision			.002
Unknown	1 (2%)	0	
Yes	47 (78%)	30 (53%)	
No	12 (20%)	27 (47%)	

**Figure 2:**
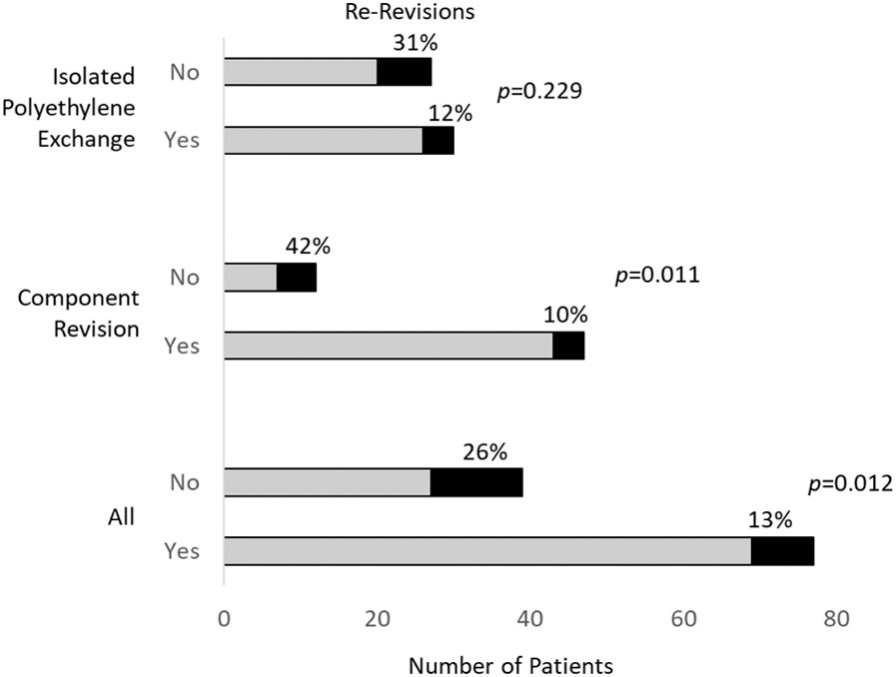
Effect of increased constraint on the proportion of patients undergoing re-revision after component revision or isolated polyethylene liner exchange (IPE). Each bar represents the number of patients who underwent revision with or without increase in the level of constraint. Black part of each bar represents the number, who subsequently required re-revision. *P* values for comparisons between patients in whom constraint did or did not increase for each treatment type, or both considered together (All).

The association between constraint levels and rate of rerevision was examined overall and in each group separately. The addition of constraint was associated with fewer rerevisions overall (Fig. 2). The rerevision rate was 31% in cases where constraint was not increased, compared to only 12% (*P* = .012) when constraint was increased. In the component revision group, rerevision was more frequent when constraint did not increase (*P* = .011); but in the IPE group, no significant effect of constraint was detected (*P* = .229, Fig. 2).

To assess functional outcomes, we compared preoperative and postoperative ROM, which was available for about half of the patients in each group (Table 5). We detected no statistically significant differences in comparing ROM between cohorts. However, there was a trend to IPE patient having more preoperatie hyperextension, which on average corrected to full extension postoperatively; as well as a trend toward less flexion in the component revision group which improved following revision.

**Table 5:** Active range of motion outcomes of patients undergoing component revision or isolated polyethylene insert exchange (IPE) for unstable Total knee arthroplasty at least 1 year postoperatively.

Motion variable	Component revision			IPE			*P* value
	Total N	Mean	SD	Total N	Mean	SD	
Extension							
Preoperative active ROM	29 (48%)	0.3	3.5	25 (44%)	−1.0	3.2	.152
Postoperative active ROM	31 (52%)	0.7	2.2	28 (49%)	0.0	0.0	.067
Delta active ROM	29 (48%)	0.3	4.0	25 (44%)	1.0	3.2	.473
Flexion							
Preoperative active ROM	29	102.9	19.3	25	111.1	22.1	.148
Postoperative active ROM	31	121.8	10.5	28	122.3	9.6	.836
Delta active ROM	29	19.4	19.7	25	10.9	22.3	.143

Postoperative KOOS JR and LEAS scores were available in at least two-thirds of patients in each group 2 years following either treatment, and mean scores in both groups were higher than the patient acceptable symptom state of 63.7 [17] (Table 6). The proportion of patients who achieved patient acceptable symptom state for KOOS JR was 55% in the component revision group and 49% in the IPE group, and this difference was not significant (*P* = .957). Similarly, no difference was detected in the number of patients achieving patient acceptable symptom state for KOOS JR when comparing those whose level of constraint did or did not increase at revision (*P* = .529), even when the type of surgery (component revision or IPE) was also considered. The mean LEAS for both groups was 10, indicative of moderate activity. Postoperative pain VAS scores were available for about half of patients in each group, and reported means did not differ based on treatment (*P* = .314).

**Table 6:** Patient-reported outcomes (PROMs) of patients 2 years after component revision or isolated polyethylene insert exchange (IPE) for unstable total knee arthroplasty.

Outcome measure	Component revision			IPE			*P* value
	Total N (%)	Mean	SD	Total N (%)	Mean	SD	
KOOSJR^a^	40 (67%)	67.2	20.5	41 (72%)	68.9	20.4	.718
LEAS^b^	40 (67%)	10	3.4	41 (72%)	10	2.5	.948
Pain VAS^c^	31 (52%)	49	26.6	32 (56%)	43	22.9	.314

a Knee Injury and Osteoarthritis Outcome Score for Joint Replacement (KJOOS JR). Interval score from 0 to 100 (higher score indicating better joint health).

b Lower Extremity Activity Scale (LEAS) for assessing activity level in patients following joint arthroplasty. Raw score from 1 to 18 (higher score indicating higher activity levels).

c Pain vvisual analog scale (VAS). Scored from 0 to 100 (0 for no pain and 100 for worst imaginable pain).

## Discussion

We retrospectively reviewed 117 patients who underwent revision for instability after TKA and found that 57 (49%) had undergone IPE. One of the surprising findings of this study is that a similar number of revisions were performed by IPE as by full component revision. This is a notable increase compared to our 2018 study, where 40% of patients underwent IPE. This alone confirms the need to better understand and document these outcomes. In our current cohort, the IPE patients had radiographs confirming appropriateness of the procedure based on an absence of gross knee malalignment. Consequently, those who underwent IPE had largely similar outcomes to those undergoing component revision. We observed no significant difference in the re-revision frequency. Infection and instability were the most common reasons for rerevision in both groups. Postoperative measures of function, including knee ROM 1 year after surgery, KOOS JR, LEAS, and pain VAS at 2 years, were similar between groups. The only difference noted in the outcomes was the effect of increased constraint, which was associated with lower rerevision in the component revision, but not IPE patients.

Instability is one of the more common reasons for revision total knee replacement, but there are no clearly defined clinical parameters for a diagnosis of instability. There is a wide variability in the states of laxity in the knee that are associated with excellent perceived clinical outcomes [18]. Yet there are clearly patients that have notable measurable laxity associated with decidedly unsatisfactory clinical performance whose clinical state improves with improved soft-tissue tension by way of a larger more constrained polyethylene [[19], [20], [21]]. The complex kinematic and soft-tissue environment around the knee provide for many different manifestations of clinical instability including coronal plane instability, sagittal plane instability, global instability, and isolated flexion instability [22]. This poses a challenge to the researcher, and to address this, we were as inclusive as possible, analyzing all consecutive cases of revision TKA with a diagnosis code indicating instability.

The comparison group of patients undergoing full component revision is an imperfect one as the indications and types of instability treated are likely only partially overlapping. For example, the proportion of patients undergoing revision due to instability of a cruciate retaining implant was very different in the 2 groups, with nearly one third (N = 18) starting with this type of implant in the component revision patients compared to only 5% (N = 3) in the IPE patients. The proportion of cases where constraint was increased at revision also differed between groups. While differences in the preoperative type of implant may have impacted whether constraint was added during revision, the decision may also have stemmed from the type or degree of instability. Nonetheless, the failure mechanisms are still likely to be comparable across the IPE and component revision patients. Indeed, the rates and reasons for rerevision were similar regardless of treatment.

Some recent studies have suggested that IPE leads to inferior outcomes when performed for instability [[13], [14], [15]], and yet in every series, there were some patients that fared well with this treatment protocol [[8], [9], [10], [11], [12], [13], [14],^16^]. It is that population that we seek to understand. In this context, the preoperative radiographic measurements of our IPE cohort are a strength of this study. These measurements reflect the clinical decision-making of the surgeons at our institution, who have reserved IPE for patients whose knees were within 3 degrees of neutral mechanical alignment with regard to the coronal plane and tibial slope (sagittal plane). In about half of our IPE cohort, we could document an absence of flexion contracture, based on preoperative ROM. The implied criteria for IPE in our study are in line with the algorithm suggested by Pang et al (2017). Although we have attempted to exclude clear cases of malalignment and significant polyethylene wear, there may be subtle elements of each factored into our cohorts such that some elements of additional pathology beyond just instability may also be part of the clinical scenarios that were treated herein [23]. For example, our film series could not exclude malrotation. Future studies providing even more detailed objective measures of knee alignment are needed before evidence-based guidelines can truly be established for the treatment of unstable TKAs.

The similarity in outcomes we observed between our component revision and IPE cohorts is consistent with previous studies [[8], [9], [10], [11]]. Despite this, the identification of the specific parameters, which make IPE the best choice for a given patient have been elusive. Tegethoff et al (2020) reported a much higher (60%) rate of reoperation within 2 years in his cohort of 20 IPE patients, even though their comparison group of 126 patients undergoing component revision had a reoperation rate similar to ours (17%). One reason for this discrepancy may be that the authors were not able to confirm appropriate alignment and fixation prior to IPE. In a large (N = 217) series of IPEs, Tetreault (2021) recently reported rerevision rates ranging between 20% (21 of 105 cases) and 67% (2 of 3 cases), depending on the reason for the IPE. In their subset of patients treated by IPE for TKA instability rerevision was higher (40 of 148 [27%]) than what we observed. However, their analysis spanned 10 years, whereas our cohort has only been followed up for 2 years so far. We are continuing to follow up our cohort to determine how our IPE patients fare over time.

The use of data from only one urban, tertiary care center is a limitation of our study. While our findings may not be projectable nationally or globally, our patient population is relatively diverse. As studies like ours are repeated elsewhere, larger trends will emerge. The short follow-up is also a significant limitation. As noted previously, the possibility of further clinical decline with time due to instability or other causes is real. We are continuing to follow up these patients for a longer-term assessment in the future.

We have not solved the controversy of IPE for TKA instability, but we have confirmed previous studies reporting similarly successful outcomes compared to component revision when patients are appropriately selected. Furthermore, we have provided a radiographic description of our IPE cohort, and this can serve as a guide for selecting patients for IPE. Given our findings, we would suggest a suitable patient for IPE would have reasonable coronal and sagittal alignment and rotation, well-fixed implants, laxity in both flexion and extension, and an implant system that will allow a constrained polyethylene to be inserted. Knees with a flexion contracture present a contraindication to IPE as any increase in polyethylene thickness will result in the worsening of the flexion contracture. The most salient limitations of our study point to the need for the development and validation of objective and subjective measures to describe TKA instability more precisely. Such metrics will make feasible future studies that can be used to establish truly evidence-based guidelines for the treatment of patients, who present with unstable TKAs.

## Conflicts of interest

José A. Rodriguez reports receiving royalties from Exactech, Inc.; is a paid employee for Exactech, Inc., Smith & Nephew, Medacta, and Conformis; received research support from Exactech, Inc. and Smith & Nephew; is a part of Clinical Orthopedics and Related Research, Journal of Arthroplasty, and HSS Journal; and is board member of American Association of Hip and Knee Surgeons (AAHKS) and Eastern Orthopedic Association (EOA). Michael Cross reports being in speakers bureau of Flexion Therapeutics amd 3M KCI; is a paid consultant for 3M KCI, Depuy Synthes, Johnson & Johnson Company, Exactech, Inc., Flexion Therapeutics, Smith & Nephew, and Intellijoint Surgical, Inc.; has stock options in Parvizi Surgical Innovation, Imagen Technologies, Intellijoint Surgical, Inc, and Insight Medical; received research support from 3M KCI, Exactech, Inc., and Intellijoint Surgical, Inc.; is a part of Medical/Orthopaedic publications editorial/governing board for Techniques in Orthopedics, Bone and Joint Journal 360, and Journal of Orthopedics and Traumatology. The other authors declare no potential conflict of interest.

For full disclosure statements refer to https://doi.org/10.1016/j.artd.2023.101134.

## Appendix A Supplementary Data

Conflict of Interest Statement for Rodriguez

Conflict of Interest Statement for Hidaka

Conflict of Interest Statement for Joseph

Conflict of Interest Statement for Sapountzis

Conflict of Interest Statement for Cross

Conflict of Interest Statement for Alamanda

Conflict of Interest Statement for Chiu
