# Supplementary material for: The Role of Constraint in Revision Total Knee Replacement for Instability: Full Component Revision Vs Isolated Polyethylene Exchange in Selected Patients
Source: Arthroplast Today. 2023 Apr 25;21:101134. doi: 10.1016/j.artd.2023.101134 (PMC10182170; doi:10.1016/j.artd.2023.101134)
Supplement: Conflict of Interest Statement for Hidaka [file mmc2.docx]

# CONFLICT OF INTEREST STATEMENT

***American Association of Hip and Knee Surgeons***

(Adopted from the American Academy of Orthopaedic Surgeons disclosure statement)

The following form **must be filled out completely and submitted by each author (example, 6 authors, 6 forms).**

**All items require a response. If there is no relevant disclosure for a given item, enter "*None*.”**

Manuscript Title: *The role of constraint in revision total knee replacement for instability: full component revision vs isolated polyethylene exchange in selected patients*

1. Royalties from a company or supplier (The following conflicts were disclosed)

none

2. Speakers bureau/paid presentations for a company or supplier (The following conflicts were disclosed)

none

3A. Paid employee for a company or supplier (The following conflicts were disclosed)

no

3B. Paid consultant for a company or supplier (The following conflicts were disclosed)

no

3C. Unpaid consultants for a company or supplier (The following conflicts were disclosed)

no

4. Stock or stock options in a company or supplier (The following conflicts were disclosed)

no

5. Research support from a company or supplier as a Principal Investigator (The following conflicts were disclosed)

no

6. Other financial or material support from a company or supplier (The following conflicts were disclosed)

no

7. Royalties, financial or material support from publishers (The following conflicts were disclosed)

no

8. Medical/Orthopaedic publications editorial/governing board (The following conflicts were disclosed)

no

9. Board member/committee appointments for a society (The following conflicts were disclosed)

no

**Each author must sign AND print or type his/her name, date and submit a separate form**

In addition, one BLINDED Conflict of Interest form (no author names used) should be submitted per manuscript with all author disclosures.

Chisa Hidaka
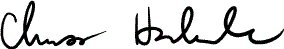
 August 1, 2022

Author Name (Print or Type) Author Signature Date
