# Supplementary material for: The Role of Constraint in Revision Total Knee Replacement for Instability: Full Component Revision Vs Isolated Polyethylene Exchange in Selected Patients
Source: Arthroplast Today. 2023 Apr 25;21:101134. doi: 10.1016/j.artd.2023.101134 (PMC10182170; doi:10.1016/j.artd.2023.101134)
Supplement: Conflict of Interest Statement for Cross [file mmc5.pdf]

INDIVIDUAL CONFLICT OF INTEREST STATEMENT

American Association of Hip and Knee Surgeons

(Adopted from the American Academy of Orthopaedic Surgeons disclosure statement)

The following form **must be filled out completely and submitted by each author (example, 6 authors, 6 forms). All items require a response. If there is no relevant disclosure for a given item, enter "*None.*"**

Manuscript Title
Does isolated polyethylene exchange in the properly selected patient for instability offer comparable outcomes to component revision total knee arthroplasty?

---

1. Royalties from a company or supplier (The following conflicts were disclosed) NONE

2. Speakers bureau/paid presentations for a company or supplier (The following conflicts were disclosed)
    - Flexion Therapeutics
    - 3M KCI

3A. Paid employee for a company or supplier (The following conflicts were disclosed) NONE

3B. Paid consultant for a company or supplier (The following conflicts were disclosed)
- 3M KCI
- Depuy Synthes, A Johnson & Johnson Company
- Exactech, Inc.
- Flexion Therapeutics
- Smith & Nephew
- Intellijoint Surgical, Inc.

3C. Unpaid consultants for a company or supplier (The following conflicts were disclosed) NONE

4. Stock or stock options in a company or supplier (The following conflicts were disclosed)
    - Parvizi Surgical Innovation
    - Imagen Technologies
    - Intellijoint Surgical, Inc.
    - Insight Medical

5. Research support from a company or supplier as a Principal Investigator (The following conflicts were disclosed)
    - 3M KCI
    - Exactech, Inc.
    - Intellijoint Surgical, Inc.

6. Other financial or material support from a company or supplier (The following conflicts were disclosed) NONE

7. Royalties, financial or material support from publishers (The following conflicts were disclosed) NONE

8. Medical/Orthopaedic publications editorial/governing board (The following conflicts were disclosed)
    - Techniques in Orthopaedics
    - Bone and Joint Journal 360
    - Journal of Orthopaedics and Traumatology

9. Board member/committee appointments for a society (The following conflicts were disclosed) NONE

Each author must sign AND print or type his/her name, date and submit a separate form

In addition, one BLINDED Conflict of Interest form (no author names used) should be submitted per manuscript with all author disclosures.

Michael Cross		July 24, 2020
Author Name (Print or Type)	Author Signature	Date
